# Molecular characterization of diverse CIMMYT maize inbred lines from eastern and southern Africa using single nucleotide polymorphic markers

**DOI:** 10.1186/1471-2164-13-113

**Published:** 2012-03-25

**Authors:** Kassa Semagn, Cosmos Magorokosho, Bindiganavile S Vivek, Dan Makumbi, Yoseph Beyene, Stephen Mugo, BM Prasanna, Marilyn L Warburton

**Affiliations:** 1 International Maize and Wheat Improvement Center (CIMMYT), P. O. Box 1041, Village Market 00621, Nairobi, Kenya; 2 CIMMYT, 12.5 Km peg Mazowe Road, P.O. Box MP163, Mount Pleasant, Harare, Zimbabwe; 3 CIMMYT, C/o ICRISAT, Patancheru 502324, India; 4 United States Department of Agriculture-Agricultural Research Service: Corn Host Plant Resistance Research Unit, Box 9555, Mississippi State, MS 39762, USA

## Abstract

**Background:**

Knowledge of germplasm diversity and relationships among elite breeding materials is fundamentally important in crop improvement. We genotyped 450 maize inbred lines developed and/or widely used by CIMMYT breeding programs in both Kenya and Zimbabwe using 1065 SNP markers to (i) investigate population structure and patterns of relationship of the germplasm for better exploitation in breeding programs; (ii) assess the usefulness of SNPs for identifying heterotic groups commonly used by CIMMYT breeding programs; and (iii) identify a subset of highly informative SNP markers for routine and low cost genotyping of CIMMYT germplasm in the region using uniplex assays.

**Results:**

Genetic distance for about 94% of the pairs of lines fell between 0.300 and 0.400. Eighty four percent of the pairs of lines also showed relative kinship values ≤ 0.500. Model-based population structure analysis, principal component analysis, neighbor-joining cluster analysis and discriminant analysis revealed the presence of 3 major groups and generally agree with pedigree information. The SNP markers did not show clear separation of heterotic groups A and B that were established based on combining ability tests through diallel and line x tester analyses. Our results demonstrated large differences among the SNP markers in terms of reproducibility, ease of scoring, polymorphism, minor allele frequency and polymorphic information content. About 40% of the SNPs in the multiplexed chip-based GoldenGate assays were found to be uninformative in this study and we recommend 644 of the 1065 for low to medium density genotyping in tropical maize germplasm using uniplex assays.

**Conclusions:**

There were high genetic distance and low kinship coefficients among most pairs of lines, clearly indicating the uniqueness of the majority of the inbred lines in these maize breeding programs. The results from this study will be useful to breeders in selecting best parental combinations for new breeding crosses, mapping population development and marker assisted breeding.

## Background

Assessment of genetic diversity, relationships, and structure within a given set of germplasm is useful in plant breeding for different reasons including: (i) assisting in the selection of parental combinations for developing progenies with maximum genetic variability for genetic mapping or further selection [1]; (ii) describing heterotic groups [2-7]; (iii) determining the level of genetic variability when defining core subsets selected for specific traits [8]; (iv) estimating possible loss of genetic diversity during conservation or selection programs [9]; and (v) estimating the relative strengths of evolutionary forces (mutation, natural selection, migration or gene flow, and genetic drift) [10,11]. In maize, the two main tasks of breeders involve the first two points, above, including developing improved inbred lines and identifying the best parental combinations for creating hybrids that are phenotypically superior and with significantly higher yield compared to their parents [12]. In species where heterosis and heterotic groups can be exploited, inbred lines are primarily developed by crossing elite lines within heterotic groups followed by inbreeding and selection, while hybrids are produced by crossing parents that belong to different heterotic groups. A heterotic group is a collection of closely related inbred lines which tend to result in vigorous hybrids when crossed with lines from a different heterotic group, but not when crossed to other lines of the same heterotic group [13]. Depending on the objectives of the breeding program, breeders use different methods in selecting the best parents for making crosses, and for assigning lines to a particular heterotic group, including (a) pedigree relationships, (b) phenotypic performance for specific traits, (c) adaptability and yield stability, (d) top crosses, (e) diallel crosses, and (f) genetic distances estimated from morphological and molecular markers [14]. Genetic distance can be estimated from various types of molecular markers, including restriction fragment length polymorphism (RFLP), amplified fragment length polymorphism (AFLP), simple sequence repeats (SSRs) and single nucleotide polymorphisms (SNPs).

Advances in molecular technology, however, have produced a shift towards SNP markers [15,16]. Because of their low cost per data point, high genomic abundance, locus-specificity, codominance, potential for high throughput analysis, and lower genotyping error rates [17-19], SNPs have emerged as a powerful tool for many genetic applications, including genetic diversity studies, linkage and quantitative trait loci (QTL) mapping, and marker-assisted breeding [20]. Currently, chip-based technology is the most high-throughput SNP genotyping platform. The Illumina chip-based SNP detection technology is useful for a broad range of applications to genotype samples with different possible levels of multiplexing, from 48 to 384 (BeadXpress) and 1536 (GoldenGate) to 55,000 SNPs (Infinium). Such chip-based genotyping platforms are suitable for large-scale studies that require genotyping of individual samples with thousands of SNPs [21]. High levels of multiplexing, high total cost and lengthy process of initial assay development are a drawback of chip-based platforms. They may be unsuitable for studies where only a small to moderate number of SNPs are needed over a large number of samples, as is the case in mapping, marker assisted recurrent selection, marker assisted backcrossing, and quality control applications. In such cases, uniplex SNP genotyping platforms are more suitable [21]. Furthermore, a significant percentage of the SNPs in highly multiplexed chip-based assays generally prove uninformative in any given population [22]. It is therefore necessary to select the best SNPs to provide a good level of discrimination for uniplex assays of each population under study.

Maize (*Zea mays *ssp. *mays *L.) is the world's third most important by acreage and is a multi-purpose crop for food, animal feed, biofuel, and raw material in the synthesis of a broad range of industrial products [23]. Over the past 4 decades, breeders at the International Maize and Wheat Improvement Center (CIMMYT), in collaboration with the National Agricultural Research Systems (NARS) of many maize-growing countries, have developed numerous germplasm pools, populations, and open-pollinated varieties [6,24,25]. CIMMYT maize germplasm is widely used by various public and private sector institutions worldwide for the development of open pollinated varieties, hybrid seed production, pedigree breeding, development of populations for QTL mapping, molecular breeding, doubled haploid production, and transgenic introduction of traits. Lu et al. [4] characterized 770 maize lines, including 394 tropical/subtropical germplasm from CIMMYT; 14 tropical/sub-tropical and 268 temperate germplasm from China; and 1 temperate and 93 tropical/subtropical germplasm from Brazil, using 1034 SNPs. The authors reported the presence of clear population structure and genetic divergence between temperate and subtropical/tropical germplasm. Yan et al. [26] studied 632 inbred lines from temperate, tropical, and subtropical public breeding programs and reported the presence of clear structure between temperate and tropical lines, and also complex familial relationships among global maize collection. Wen et al. [27] studied an association mapping panel consisting of 359 maize inbred lines both from CIMMYT and International Institute for Tropical Agriculture (IITA) breeding programs that have resistance to drought, low nitrogen, soil acidity, pest and disease resistance. The authors reported the presence of a subgroup that largely consisted of lines developed from LaPosta Sequía. All the previous three studies, however, included some of the maize inbred lines that were either developed by the CIMMYT maize breeding programs in eastern and southern Africa or widely used CIMMYT Maize Lines (CMLs) in the region. The main objective of our study was to investigate the population structure and patterns of relationships of the maize inbred lines from CIMMYT maize improvement programs in Zimbabwe and Kenya for better exploitation in breeding programs. The other objectives of our study were to assess the utility of SNPs in classifying maize inbred lines into one of two heterotic groups commonly used by the CIMMYT breeders, and identify a subset of highly informative SNP markers for routine and low cost genotyping of CIMMYT germplasm in the region.

## Methods

A total of 450 maize lines (382 fixed inbred parental lines and 68 advanced breeding lines) from CIMMYT breeding programs in Kenya and Zimbabwe were used in this study (Additional file 1: Table S1). The germplasm included: (a) drought tolerant inbred and advanced breeding lines developed for sub Saharan Africa (SSA) and used by breeders; (b) other parental lines used mainly by breeders in SSA for starting new line pedigree populations, mapping population development, trait introgression through transformation, double haploid production, and marker assisted recurrent selection; (c) elite breeding lines and released CMLs used to form hybrids that are evaluated in CIMMYT regional trials across eastern, southern, and central Africa, and (d) quality protein maize (QPM) inbred lines. DNA was extracted from greenhouse grown seedlings at the 3-4 leaf stage at the Biosciences for eastern and central Africa (BecA) hub in Nairobi, Kenya, using a modified version of CIMMYT protocol http://www.generationcp.org/capcorner/chile_wksp_2005/manuals/manual_01.pdf. Normalized DNA was transferred to 96-well plates and shipped to the Cornell University Life Sciences Core Laboratories for genotyping. Samples were genotyped with the 1536 random SNP chip [4] using an Illumina BeadStation 500 G (Illumina, San Diego, CA, USA) as described elsewhere [28]. Alleles were called on data from combined plates with the Illumina BeadStudio version 3.0 genotyping software and checked manually when errors were observed in known homozygote and heterozygote genotypes. For each SNP, number of alleles, allele frequency, number of genotypes, genotype frequency, observed heterogeneity, gene diversity, and polymorphic information content (PIC) were computed using PowerMarker version 3.25 [29].

An admixture model-based clustering method was used to infer population structure of the 450 lines using the software package STRUCTURE, version 2.3.3 [30]. STRUCTURE was run by varying the number of clusters (K) from 1 to 10; each K was run 3 times with a burn-in period of 100,000 and 100,000 MCMC (Markov Chain Monte Carlo) replications after burn-in. Allele frequencies were assumed to be correlated and loci were assumed to be unlinked. Individuals with probability of membership ≥ 60% were assigned to the same group while those with < 60% probability memberships in any single group were assigned to a "mixed" group [4,31]. The most probable value of K was estimated using the ad hoc statistic ΔK [32], which is based on the second order rate of change of P(X|K), the posterior probability of the data with respect to a given K. A stepwise forward discriminant analysis was run as described elsewhere [33] using XLSTAT 2010 (Addinsof, New York, USA; http://www.xlstat.com) to select an optimal set of discriminating SNPs that tended to separate the groups obtained from population structure analysis to a maximum degree. Variables (SNPs) were chosen to enter or leave the discrimination model among groups based on the significance level of an F-to-enter and F-to-remove value of 0.05 and 0.10, respectively.

Matrices of Roger's genetic distance [34] and relative kinship were calculated between each pair of lines in the study using PowerMarker [29] and TASSEL [35] software, respectively. Groups of closely related lines tend to bring redundant values to a breeding program, and a set of genetically unique lines can be chosen based on marker information. A dendrogram was constructed from the genetic distance matrix using the neighbor-joining algorithm with PowerMarker and the resulting trees were visualized using MEGA version 5.0 [36]. Principal component analysis (PCA) was performed using the software JMP version 7.0 (SAS Institute Inc., Cary, NC, USA). The first two principal components were plotted for visual examination of the clustering pattern of lines. Finally, analysis of molecular variance (AMOVA) was used to partition the variation among and within group (population) components [37]. For AMOVA, the individuals were assigned into populations using the results from population structure analysis, cluster analysis and *a priori *heterotic groups assigned to a subset of 220 lines by CIMMYT breeders. Significance levels for variance component estimates were computed using 1000 permutations. Both AMOVA and F_ST _were calculated using the ARLEQUIN version 3.11 http://cmpg.unibe.ch/software/arlequin3.

## Results

### SNP characteristics

**Figure 1:**
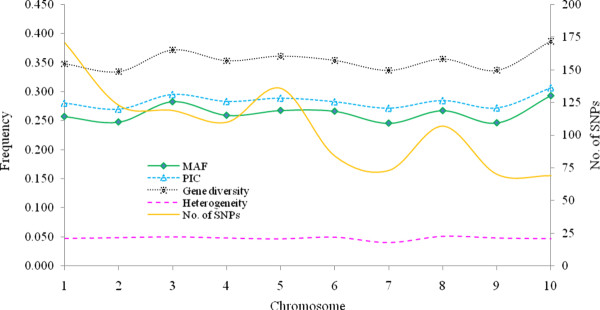
**Summary of the 1065 SNPs used for genotyping the 450 maize inbred lines from CIMMYT breeding programs in eastern and southern Africa**. Minor allele frequency (MAF), heterogeneity, gene diversity (GD) and polymorphic information content (PIC) are averages of all SNPs per chromosome.

Of the 1536 SNPs, 471 (30.7%) were not included in data analyses because they showed weak amplification, ambiguity or irreproducibility in allele calling, high (> 10%) missing data, or had a minor allele frequency (MAF) of less than 5%. Figure 1 summarizes the 1065 SNPs, including number of markers, heterogeneity, gene diversity, minor allele frequency (MAF) and PIC per chromosome; a more detailed table of this data can be found in Additional file 2: Table S2. Roughly one third of the SNPs (37.7%) showed a MAF between 0.051 and 0.200 (relatively infrequent), and 32.3% had a PIC value ≤ 0.25.

### Population structure

**Figure 2:**
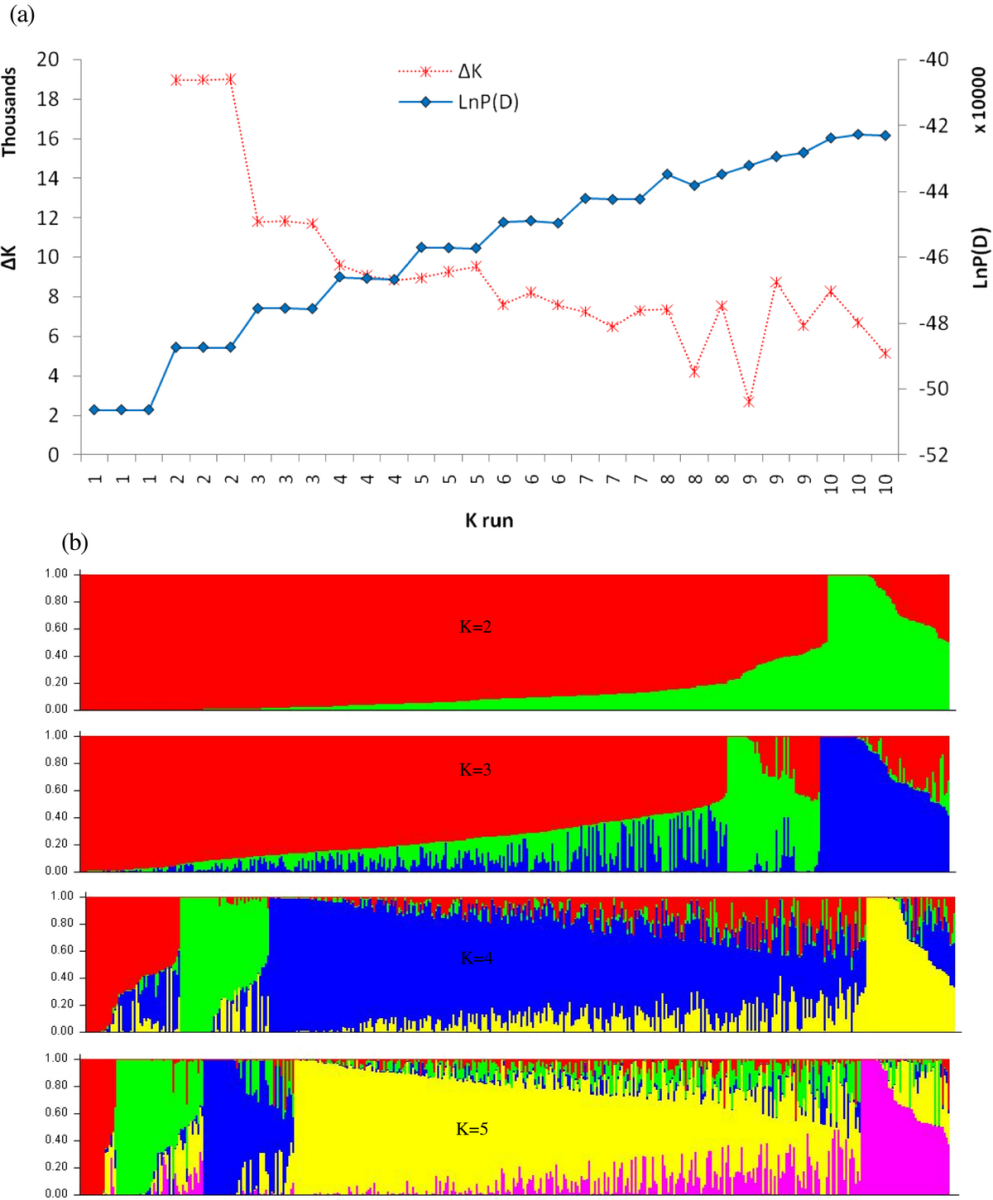
**Population structure of 450 maize inbred lines based on 1065 SNP markers: (a) plot of LnP(D) and an ad hoc statistic ΔK calculated for K ranging from 1 to 10, with each K repeated trice; (b) population structure of the 450 lines between K = 2 to K = 5**. Each individual is represented by a single vertical line that is partitioned into K colored segments (K = 2 to K = 5) in the x-axis, with lengths proportional to the estimated probability membership (y-axis) to each of the K inferred clusters.

The estimated log probability of the data (LnP(D)) increased continuously with increasing K (number of groups or populations) and there was no obvious K value clearly defining the number of populations. However, LnP(D) sharply increased between K = 1 and K = 3, and much less so between K = 4 and K = 10 (Figure 2a). The ad hoc statistic ΔK showed a higher likelihood values at K = 2, with a sharp decrease when K increased from 3 to 4 (Figure 2a). When the results from different K values were compared with pedigree and breeding history, the groups obtained at K = 3 (Figure 2b) seem the best possible number of populations, and 83.1% of the lines were assigned into one of the populations at this number of clusters (Additional file 1: Table S1). The majority of the lines (291) were assigned to group 1, which included 101 maize streak virus (MSV) resistant lines, 58 QPM lines (15 of which were extracted from QPM population POOL15), 16 weevil resistant lines, 22 drought tolerant lines (most of them from population La Posta Sequia), 24 multiple borer resistant (MBR) lines extracted from cycles 3 and 5 of MBR population, and 6 MSV resistant lines from populations 100 and 300. The second group consisted of 31 lines of which about 90% had at least one maize streak resistant parent (either CML202 or OSU23i from Ohio State University) in their pedigree. Group 3 consisted of 52 lines that are MSV resistant, 14 of which have CML390 as a common parent. Breeding for maize streak virus resistance has been one of the major efforts in the CIMMYT maize breeding program based in Zimbabwe. The remaining 76 genotypes were classified into a mixed group as they had membership probabilities < 60% to be assigned into one of the three groups.

**Figure 3:**
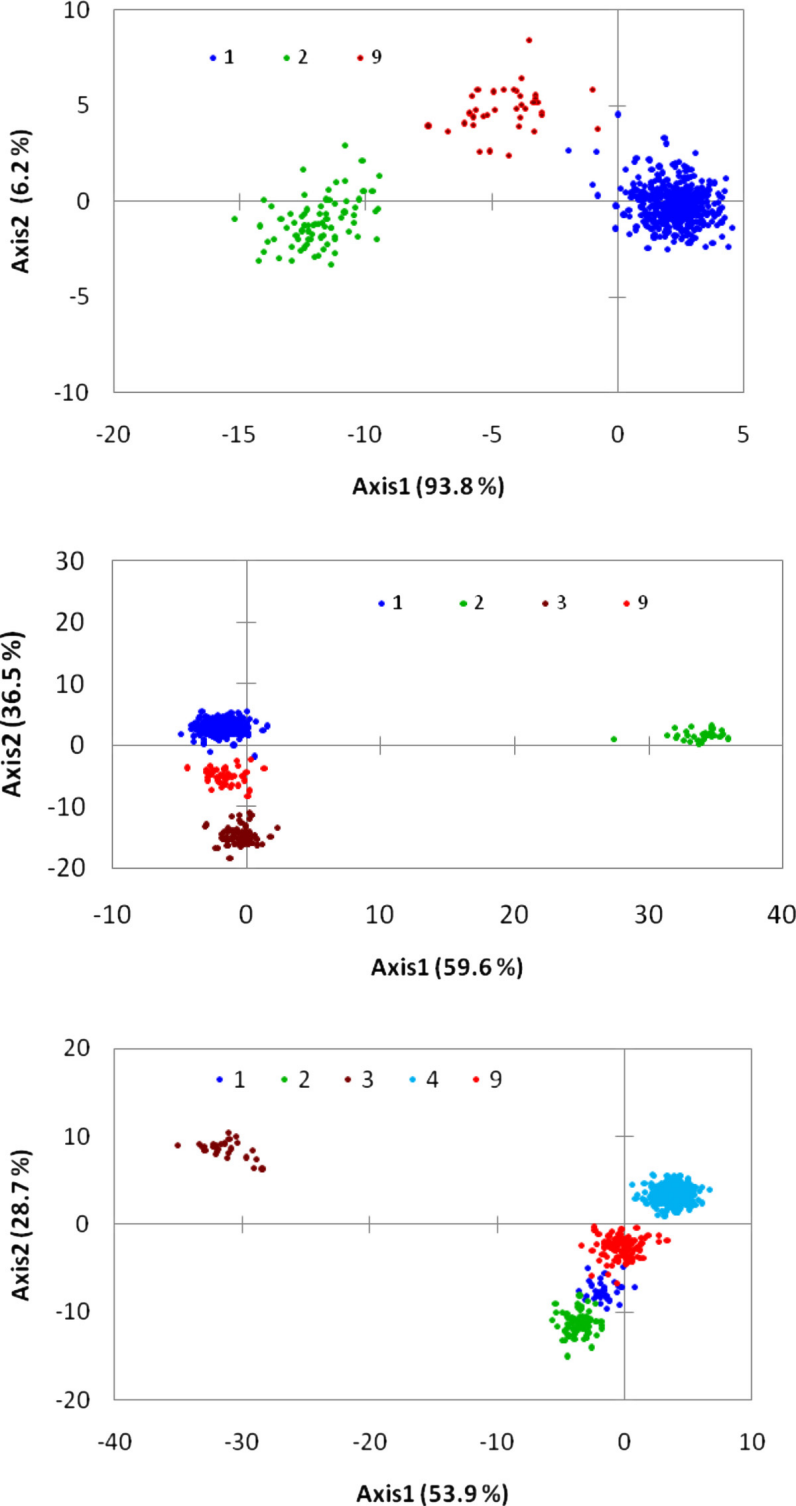
**Plot of Axis-1 and Axis-2 from discriminant analyses using *a priori* information obtained from population structure analyses at K = 2 (top), K = 3 (middle) and K = 4 (bottom)**. The numbers 1, 2, 3, 4 and 9 refer to population 1, population 2, population 3, population 4 and mixed group, respectively.

In order to assess the reliability of the different groups obtained through the model-based population structure analyses, we ran discriminant analyses using the groups obtained from population structure as categorical variables. The discriminant analyses clearly separated the populations and mixed group obtained both at K = 2 and K = 3. When the population structure obtained from K = 4 to K = 10 were used as categorical variables in the discriminant analyses, the results remained basically same as the one obtained at K = 3, and only 3 populations and a mixed group were clearly visible in the plot. The discrimination model with the stepwise procedure identified 237 alleles from 236 SNPs as the best explanatory variables for the *priori *group defined at K = 3. When the *priori *group at K = 3 were used in plotting the two axes from discriminant analyses, axis-1 separated group 2 from groups 1, group 3 and the mixed group (Figure 3). Axis-2 further separated group 1 from group 3, with the mixed group being intermediate between them. Fisher and Mahalanobis distance matrices from pairwise comparisons of the three groups and mixed group were all significant, with group 2 being at least 4 to 12 times more distant from all others. The correct classification of lines into their respective population, based on the 236 selected SNPs, was 99.8%.

### Genetic distance and relationship

**Figure 4:**
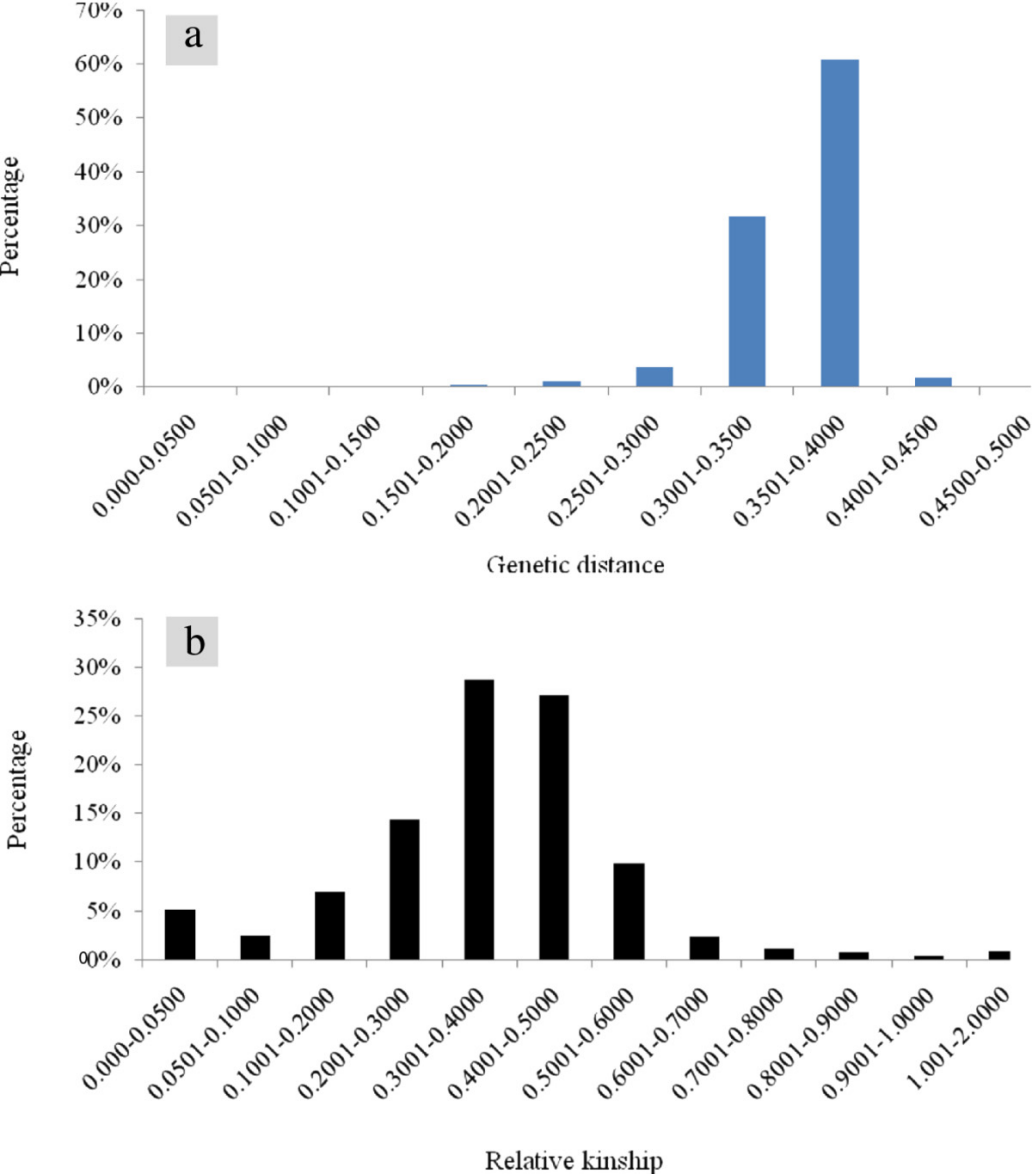
**Distribution of pairwise (a) Roger's genetic distance and (b) relative kinship calculated for 450 maize inbred lines genotyped with 1065 SNPs**. Relative kinship values close to 0 indicate no relationship.

**Figure 5:**
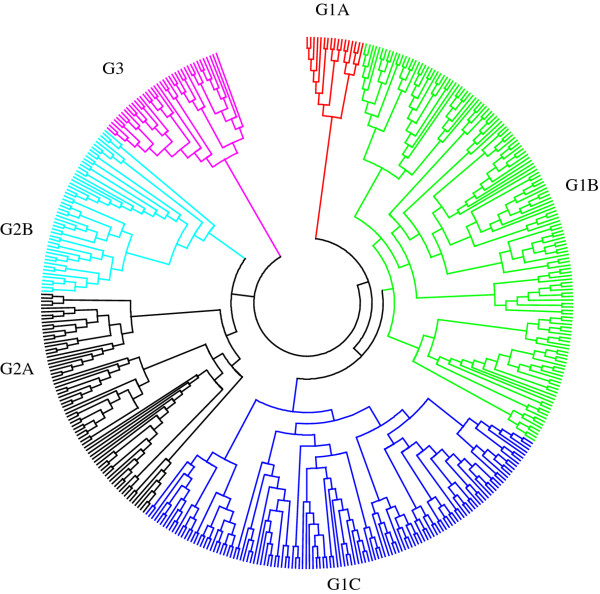
**Neighbor-joining tree for 450 inbred lines based on Roger's genetic distance calculated from 1065 SNP markers**. The subgroups are indicated with different color and a detail of the different subgroups is given in additional file 1:Table S1.

**Figure 6:**
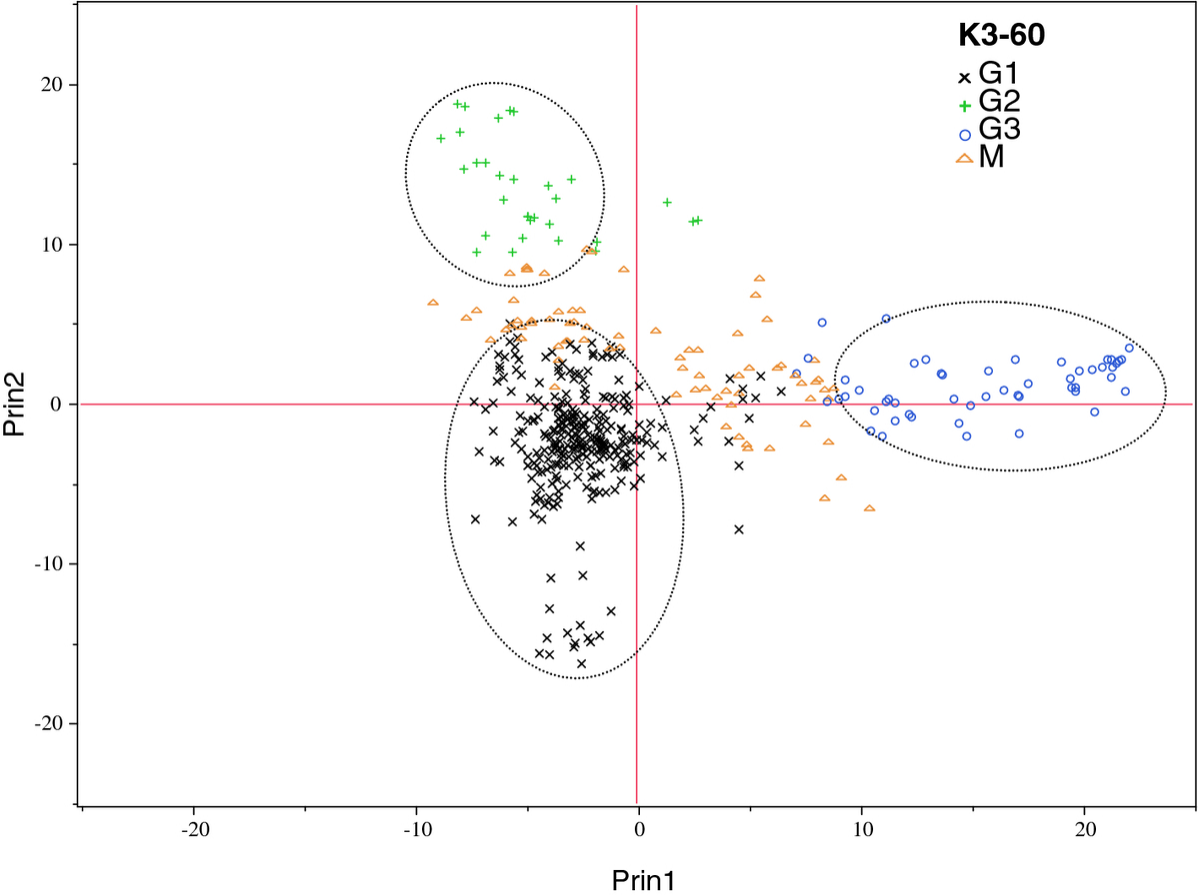
**Principal component (PC) analysis of 450 maize germplasm genotyped with 1065 SNPs**. PC1 (5.3%) and PC2 (3.4%) separated the lines into 3 major groups. The groups from PCA supports the presence of population structure at K = 3. Individuals that were assigned in to a mixed group in the population structure analysis are indicated in red color.

Roger's genetic distance between pairwise comparisons of all the 450 lines ranged from 0.003 to 0.450, and the overall average distance was 0.353; however, the vast majority (94.2%) fell between 0.300 and 0.400 (Figure 4a). Relative kinship coefficients between pairs of samples varied from 0 to 1.97 (Figure 4b), with an overall average of 0.370, but most (79%) values were from 0.050 to 0.500. The neighbor-joining (NJ) tree generated from Roger's genetic distance matrix grouped the 450 lines into 3 major groups and 6 subgroups (Figure 5; Additional file 1: Table S1). Group 1 consisted of 288 very diverse lines, including early maturing lines (1A); MSV resistant, extra-early, and QPM lines (1B); and MSV resistant lines (1 C). Group 2 consisted of 123 lines, including 16 weevil resistant, 24 multiple borer resistant and 18QPM lines (2A). Nearly half (25 out of 51) of the lines in group 2B were QPM lines. Group 3 consisted of mainly drought tolerant lines from La Posta Sequia population, MSV resistant lines extracted from populations 100 and 300, and some early-intermediate maturing lines. There was low concordance between the neighbor-joining clustering and model-based population partition in assigning lines into the different groups or populations. The first two principal components (PCs) from principal component analysis explained 8.7% of the total SNP variations among samples. A plot of PC1 (5.3%) and PC2 (3.4%) revealed 4 major groups (Figure 6) and the pattern of groupings was basically the same as that of the model-based population partition at K = 3. Nearly all individuals assigned to a population at K = 3 were in the same group in the principal component analysis, with the mixed group being intermediate between the 3 populations.

### Heterotic grouping

**Figure 7:**
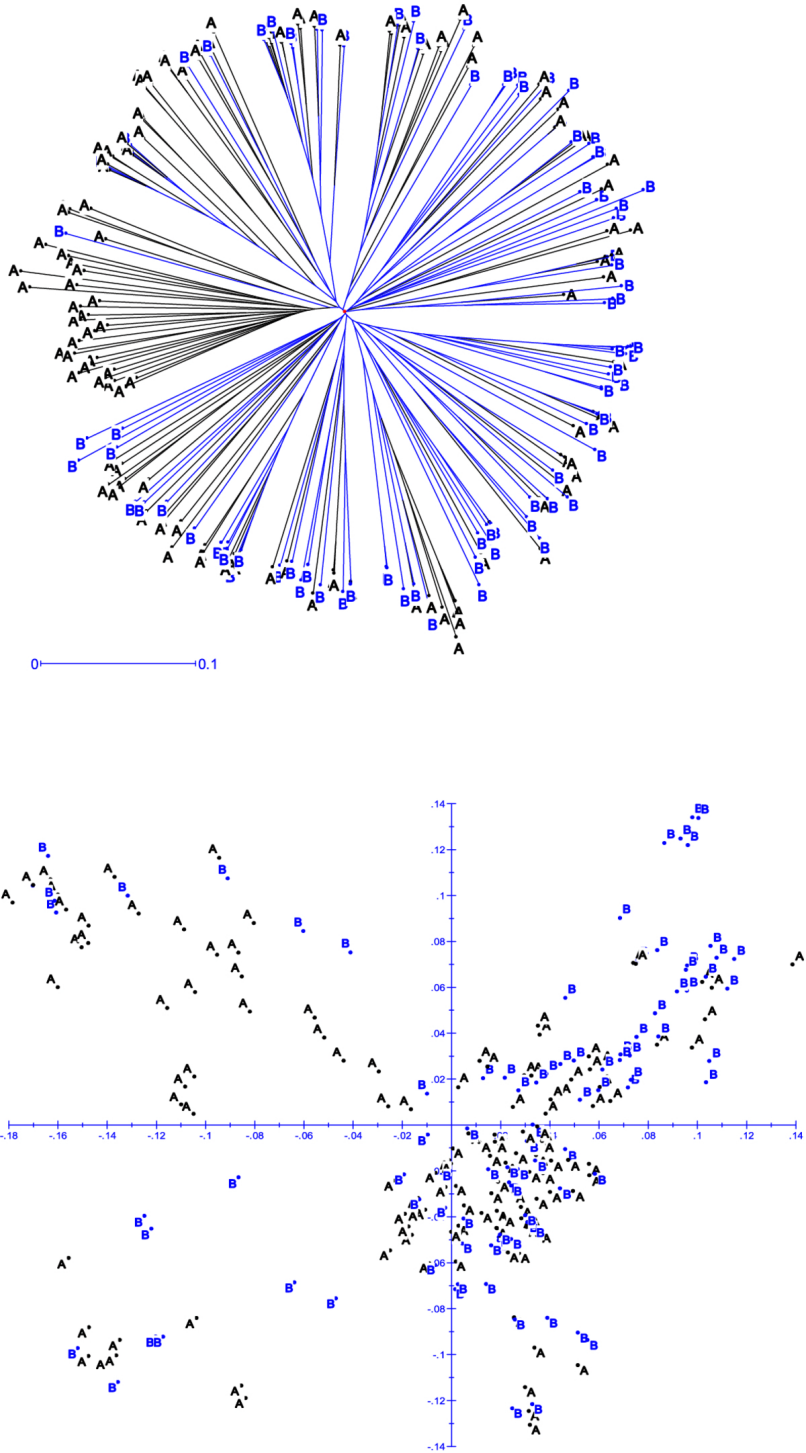
**Cluster (top) and principal component (bottom) analyses of 220 inbred lines that were assigned in to heterotic groups A and B based on combining ability tests through diallel and line x tester analyses**. A and B refers to heterotic group A and B.

In order to assess if the SNP data show clear genetic differentiation between CIMMYT heterotic groups A and B that were established based on combining ability tests through diallel and line x tester analyses, we ran population structure analyses at both K = 2 and K = 3, and NJ-clustering and principal component analyses by selecting a subset of 220 out of the 450 lines that belong to heterotic group A (126 lines) and B (94 lines). At both K = 2 and K3, there was very little correspondence between heterotic grouping based on phenotypic data and the model-based population partition based on the SNP data (Additional file 3: Table S3). For example, at K = 3, population structure analysis assigned 26.2% of the lines from heterotic group A and 12.8% of the lines from heterotic group B into group 1; 4.8% of the lines from heterotic group A and 7.4% of the lines from heterotic group B into group 2; 31.7% of the lines from heterotic group A and 44.7% of the lines from heterotic group B in to group 3, and the remaining 37.3% of the lines from heterotic group A and 35.1% of the lines from heterotic group B in to a mixed group. Similar results were obtained with K = 2. Both NJ-clustering and principal component analyses did not show a clear pattern in separating the 220 lines into heterotic groups (Figure 7).

### AMOVA

Table 1 shows the partitioning of the overall SNP variance into hierarchical levels using AMOVA. When AMOVA was performed using the 3 and 6 possible groups predicted from NJ-cluster analyses, the estimated fixation indices (*F_ST_*) varied from 3.9% to 6.8%. When the overall SNP variance was partitioned into hierarchical levels using the groups predefined from the model-based population partition at K = 2, K = 3, K = 4, K = 5 and K = 6 as categorical variables, F_ST _accounted for 11.7%, 9.8%, 11.1%, 12.6% and 14.0%, respectively. A random permutation test indicated that the proportion of variances attributable at all groups were highly significant (p < 0.0001). We also computed F_ST _values using the two heterotic groups created based on *a priori *knowledge of combining ability from field experiments and the groups predefined based on population structure analyses both at K = 2 and K = 3. The F_ST _was the lowest (1.2%) for heterotic groups created based on field experiments and higher for groups defined based on population structure analyses of the SNP data (F_ST _= 11.3% at K = 2 and 11.8% at K = 3).

**Table 1:** Analysis of molecular variance (AMOVA) for the extraction of SNP variation among groups (populations) and among individuals within populations

Groups	Source of variation	**d.f**.	Sum of squares	Variance components	Percentage of variation
K = 2 (2 pops and mixed)	Among populations	2	6788.84	23.91	11.74*
	Within populations	897	161224.47	179.74	88.26
	Total	899	168013.30		

K = 3 (3 pops and mixed)	Among populations	3	9812.27	19.27	9.84*
	Within populations	896	158201.03	176.56	90.16
	Total	899	168013.30		

K = 4 (4 pops and mixed)	Among populations	4	12807.29	21.69	11.12*
	Within populations	895	155206.01	173.41	88.88
	Total	899	168013.30	195.10	

K = 5 (5 pops and mixed)	Among populations	5	15428.74	24.69	12.64*
	Within populations	894	152584.56	170.68	87.36
	Total	899	168013.30	195.36	

K = 6 (6 pops and mixed)	Among populations	6	17184.95	27.57	14.03*
	Within populations	893	150828.35	168.90	85.97
	Total	899	148013.30	196.47	

Cluster analyses -3 groups	Among populations	2	3729.07	7.35	3.86*
	Within populations	897	164284.23	183.15	96.14
	Total	899	168013.30	190.50	

Cluster analyses-5 groups	Among populations	4	7377.54	10.08	5.32*
	Within populations	895	160635.77	179.48	94.68
	Total	899	168013.30	189.56	

Cluster analyses-6 groups	Among populations	5	9832.12	12.90	6.80*
	Within populations	894	158181.18	176.94	93.20
	Total	899	168013.30	189.84	

Heterotic groups (K = 2)	Among heterotic groups	2	4281.98	22.46	11.29*
	Within heterotic groups	437	77179.51	176.61	88.72
	Total	439	81461.49		

Heterotic groups (K = 3)	Between heterotic groups	3	7354.74	22.76	11.81*
	Within heterotic groups	436	74106.75	169.97	88.19
	Total	439	81461.49	192.73	

Heterotic groups-breeders (A, B)	Between heterotic groups	1	676.51	2.29	1.22*
	Within heterotic groups	438	80784.98	184.44	98.78
	Total	439	81461.49	186.73	

*p-value < 0.0001

## Discussion

### Population structure and genetic relationship

The main challenges in analyzing any genetic dataset are to (a) explore whether a given population is homogeneous or contains genetically distinct subgroups, and (b) identify quantitative evidence that supports the presence of these groups [38]. Using SNP markers, we investigated the extent of genetic differentiation, population structure, and patterns of relationship among a diverse set of maize inbred lines using the model-based population structure analysis, NJ-cluster analysis, principal component analysis, and discriminant analysis. All these different multivariate methods revealed the presence of 3 major possible groups, which was in general agreement with pedigree information, as most lines with similar pedigree tended to cluster into the same group. Our results, however, did not show clear separation by breeding programs, maturity groups (extra early, early, intermediate and late), adaptation or mega-environments (lowland tropical zone, wet and dry subtropical zones and highland zone), and specific traits such as disease resistance (e.g, resistance to maize streak virus), and drought tolerance. Our results generally agree with previous studies [39,40] who reported lack of clear clustering patterns of CIMMYT germplasm based on phenotypes, environmental adaptation, grain color or type, maturity and heterotic groups.

Comparisons of the different multivariate analyses revealed high concordance among the PCA, model-based population partition and discriminant analyses in terms of the number of groups and members of each group. However, cluster analysis showed low concordance with the other methods in terms of assigning genotypes into their respective groups (Additional file 1: Table S1). Population structure analysis was used to classify individuals into groups based on a genetic model, whereas discriminant analysis was used to summarize variation between *priori *predefined groups based on population structure so the agreement between these two methods is not unexpected. In cluster analysis, different combinations of genetic distance/similarity matrix and clustering algorithm can give rise to somewhat different groups. A single distance matrix and a single clustering algorithm even may produce several alternative clusters that often create ambiguity in selecting the best one. Because PCA produces a 2- or 3-dimensional scatter plot of the samples in which geometrical distances among samples in the plot reflect the genetic distances among them with a minimum of distortion and ambiguity compared to cluster analysis [41], we think that the pattern of grouping from PCA, population structure analysis and discriminant analysis is more reliable than the NJ-clustering in the present study.

Among the 101,025 pairwise comparisons of the 450 lines, only 0.1% fell within a genetic distance less than 0.05, indicating a lack of redundant lines among the germplasm studied. Kinship analyses agree, since kinship coefficients for 84% of the pairs of lines fell below 0.50. This suggests that each line in the study is potentially contributing new alleles to a breeding program. Our results on kinship are higher than that of Wen et al. [27] who reported pairwise kinship values close to zero for about 60% of 359 inbred maize lines. The *F_ST _*values for the *prior *groups defined here based on results from population structure analysis (0.098 to 0.140) indicated a moderate level of genetic differentiation. F_ST _values were the lowest (0.039-0.068) when the *prior *groups from cluster analyses were used as categorical variables, suggesting little to moderate levels of differentiation. Similar F_ST _values have also been reported elsewhere [27].

### Heterotic groups

Maize breeders generally develop new parental inbred lines by selecting the progeny of intercrossed lines from within the same heterotic group. As heterotic group assignment is made based on combining ability from diallel experiments, several authors suggested the use of molecular markers in heterotic grouping [2-7]. However, the SNP data in the present study (Table 1; Figure 7) did not reveal clear population structure and genetic differentiation for most inbred lines in heterotic groups A and B, as defined by CIMMYT breeders. This is in agreement with previous reports that showed no clear heterotic patterns in subtropical and tropical CIMMYT maize inbred lines [4,24,27,39,40]. Temperate maize inbred lines are developed using advanced cycle pedigree breeding by making crosses within the same pool of elite lines, leading to clearly defined groups with maximum genetic distance between groups and minimum distance within groups. CIMMYT breeders initially used broad based pools and populations to develop open pollinated varieties (OPV). CIMMYT populations are selected via modified full or half-sib recurrent selection to have high yield potential and yield stability under a wide variety of production conditions and environments in the developing world [42]. To exploit hybrid technologies, assignment of CIMMYT populations and inbred lines into heterotic groups via crossing to various representative testers has been intensified since the early 1990s. It is challenging to divide lines into heterotic groups when many lines were developed from the same original pool without regard to racial origin or heterotic pattern. Furthermore, selection within each heterotic group is not very advanced, and maximum heterotic response between groups has not yet been achieved [24]. Therefore, many generations of reciprocal recurrent selection (RRS) may be necessary before the lines from each heterotic group begin to be significantly diverged [40].

### SNP selection

Reproducibility, polymorphism frequencies, and ease of scoring are important criteria to evaluate the value of markers for germplasm characterization. Just over 30% of the 1536 SNPs scored in this study did not meet these criteria for this set of germplasm, and none of the 1065 SNPs were found to be highly informative based on PIC; this was not unexpected given the biallelic nature of SNPs. In order to recommend a useful subset of SNPs for routine genetic diversity and mapping studies in tropical and sub-tropical maize germplasm (those related to the materials in this study) using uniplex assays, we chose 644 of the most informative and repeatable SNPs (listed in Additional file 2: Table S2). The correlation between the Roger's genetic distance calculated from the 644 and the 1065 SNPs was very high in this study (r = 0.937; p < 0.0001), indicating that the information gained from these other 421 SNPs is redundant. Uniplex assays are suitable for studies where only a small to moderate number of SNPs are needed, as is the case in mapping, marker assisted recurrent selection, marker assisted backcrossing, and quality control applications. Lu et al. [4] recommended 449 out of 1034 SNPs that were found to be the best for the detection of genetic diversity in temperate, subtropical and tropical maize germplasm with least preferences to temperate lines. Three hundred fifty eight out of the 644 SNPs (55.6%) that we recommend for routine genetic diversity and mapping studies in tropical and sub-tropical CIMMYT maize germplasm using uniplex assays were common between the two studies. We have found outsourcing SNP genotyping to commercial service providers an economical and convenient option, with the most commonly used platform for low to medium marker density being the uniplex assay of Kbioscience http://www.kbioscience.co.uk. For other applications that require high density and lower cost genotyping per data point, genotyping-by-sequencing [43] is likely to take over in the near future with a cost of about $20 per DNA sample, generating over half a million SNPs http://www.maizegenetics.net/gbs-overview. CIMMYT is collaborating with Cornell University and the USDA Agricultural Research Service in implementing the genotyping-by-sequencing pipeline for genomic selection to reduce the genotyping costs below that of field phenotyping.

## Conclusions

There were high genetic distance and low kinship coefficients among most pairs of lines, clearly indicating the uniqueness of the majority of the inbred lines in these maize breeding programs. In the different multivariate analyses, several lines with similar pedigree often clustered into the same group, but the groups did not correspond to breeding programs, maturity groups or adaptation. There was no correlation between heterotic grouping based on phenotypic and SNP data. About 40% of the SNPs in the multiplexed chip-based GoldenGate assays were uninformative in this study and we recommend 644 of the 1065 for low to medium density genotyping in tropical maize germplasm using uniplex assays. The results from this study will be useful to breeders in selecting best parental combinations for starting new pedigree populations, mapping population development and marker assisted breeding.

## Competing interests

The authors declare that they have no competing interests.

## Authors' contributions

KS was responsible for DNA preparation, data scoring and analyses, and writing the manuscript. CM, BSV, DM, YB and SM selected the inbred lines for the study and assisted in germplasm characterization during manuscript preparation. BMP and MLW contributed to and edited the manuscript. All authors have read and approved the final manuscript.

## Supplementary Material

Additional file 1 **Table S1 Germplasm summary**. Click here for file

Additional file 2 **Table S2 SNP summary**. Click here for file

Additional file 3 **Table S3 Population structure summary for heterotic groups**. Click here for file
